# Glycaemic patterns during breastfeeding with postpartum use of closed-loop insulin delivery in women with type 1 diabetes

**DOI:** 10.1007/s00125-024-06227-z

**Published:** 2024-07-19

**Authors:** Lois E. Donovan, Rhonda C. Bell, Denice S. Feig, Patricia Lemieux, Helen R. Murphy, Ronald J. Sigal, Josephine Ho, Heidi Virtanen, Susan Crawford, Jennifer M. Yamamoto

**Affiliations:** 1 Department of Medicine, Cumming School of Medicine, University of Calgary, Calgary, AB Canada; 2 Department of Obstetrics and Gynecology, Cumming School of Medicine, University of Calgary, Calgary, AB Canada; 3 Alberta Children’s Hospital Research Institute, Calgary, AB Canada; 4 Division of Human Nutrition, Department of Agricultural, Food, and Nutritional Sciences, University of Alberta, Edmonton, AB Canada; 5 Department of Medicine, University of Toronto, Toronto, ON Canada; 6 Lunenfeld-Tanenbaum Research Institute, Sinai Health System, Mount Sinai Hospital, Toronto, ON Canada; 7 Department of Medicine, University Laval, Quebec City, QC Canada; 8 Norwich Medical School, University of East Anglia, Norwich, UK; 9 Department of Cardiac Sciences, Cumming School of Medicine, University of Calgary, Calgary, AB Canada; 10 Department of Community Health Sciences, Cuming School of Medicine University of Calgary, Calgary, AB Canada; 11 Department of Pediatrics, Division of Endocrinology, Cumming School of Medicine, University of Calgary, Calgary, AB Canada; 12 Alberta Perinatal Health Program, Alberta Health Services, Calgary, AB Canada; 13 Department of Internal Medicine, University of Manitoba, Winnipeg, MB Canada; 14 Children’s Hospital Research Institute of Manitoba, Winnipeg, MB Canada

**Keywords:** Breastfeeding, Closed-loop insulin delivery, Continuous glucose monitoring, Hypoglycaemia, Insulin pump therapy, Type 1 diabetes

## Abstract

**Aims/hypothesis:**

This study aimed to describe the relationship between breastfeeding episodes and maternal glucose levels, and to assess whether this differs with closed-loop vs open-loop (sensor-augmented pump) insulin therapy.

**Methods:**

Infant-feeding diaries were collected at 6 weeks, 12 weeks and 24 weeks postpartum in a trial of postpartum closed-loop use in 18 women with type 1 diabetes. Continuous glucose monitoring (CGM) data were used to identify maternal glucose patterns within the 3 h of breastfeeding episodes. Generalised mixed models adjusted for breastfeeding episodes in the same woman, repeat breastfeeding episodes, carbohydrate intake, infant age at time of feeding and early pregnancy HbA_1c_. This was a secondary analysis of data collected during a randomised trial (ClinicalTrials.gov registration no. NCT04420728).

**Results:**

CGM glucose remained above 3.9 mmol/l in the 3 h post-breastfeeding for 93% (397/427) of breastfeeding episodes. There was an overall decrease in glucose at nighttime within 3 h of breastfeeding (1.1 mmol l^−1^ h^−1^ decrease on average; *p*=0.009). A decrease in nighttime glucose was observed with open-loop therapy (1.2 ± 0.5 mmol/l) but was blunted with closed-loop therapy (0.4 ± 0.3 mmol/l; *p*<0.01, open-loop vs closed-loop).

**Conclusions/interpretation:**

There is a small decrease in glucose after nighttime breastfeeding that usually does not result in maternal hypoglycaemia; this appears to be blunted with the use of closed-loop therapy.

**Graphical Abstract:**

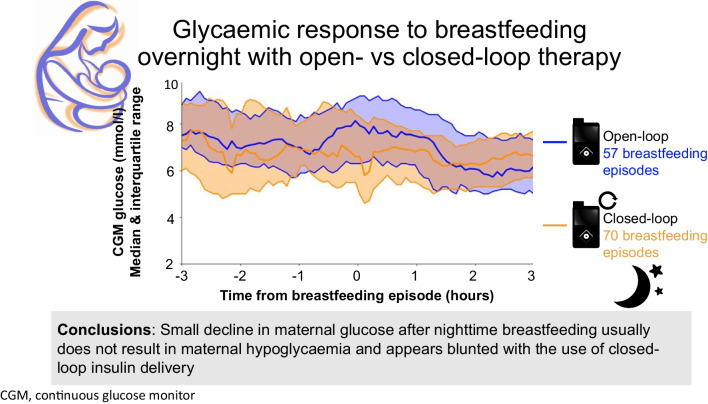


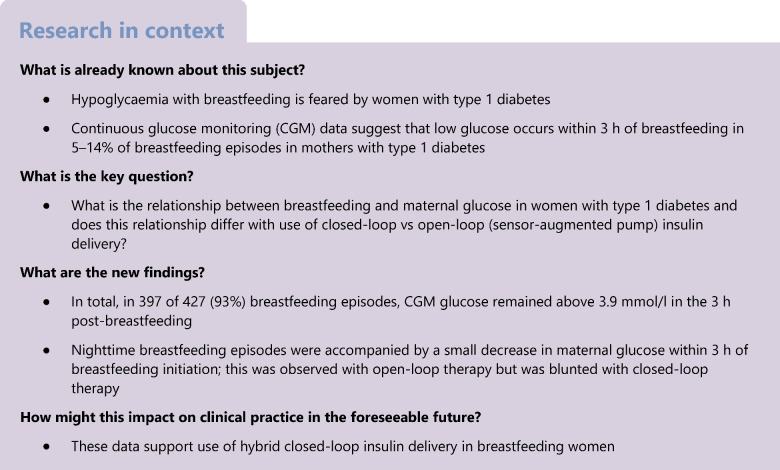


## Introduction

Breastfeeding is recommended in women with diabetes because of the associated short- and long-term benefits to the child, including reduced risk of obesity [1, 2] and diabetes [3, 4]. Despite this, women with diabetes are less likely to breastfeed than those without diabetes [5]. While the reasons for this are likely multifactorial, hypoglycaemia following breastfeeding is a feared event among women with type 1 diabetes [6].

This exploratory study examines the relationship between maternal interstitial continuous glucose monitoring (CGM) glucose levels and breastfeeding episodes. Data were collected from infant-feeding diaries obtained during the CLIMB study, which was a randomised controlled trial of closed-loop insulin delivery postpartum [7].

The aim of this study was to describe the relationship between breastfeeding episodes and maternal glucose, and to assess if this relationship differs according to postpartum insulin therapy with closed-loop or open-loop (sensor-augmented pump) therapy.

## Methods

This study represents an exploratory secondary analysis of data collected during the CLIMB study, which was a randomised trial (ClinicalTrials.gov registration no. NCT04420728)

### CLIMB study

Design details of the CLIMB study have previously been reported [7]. Briefly, the CLIMB study was a postpartum randomised controlled trial of closed-loop insulin delivery with the MiniMed 670G/770G systems (Medtronic, Northridge, CA, USA). Eighteen women used the MiniMed 670G/770G insulin pump with real-time CGM (Guardian Link 3 sensors and transmitters; Medtronic) from 1 week to 24 weeks postpartum; from 1 week to 11 weeks and 6 days postpartum, participants were randomised 1:1 to use either closed-loop (MiniMed 670G/770G in ‘Auto Mode’) or open-loop (MiniMed 670G/770G in ‘Manual Mode’) insulin delivery. During a continuation phase, all CLIMB participants were assigned to used closed-loop therapy from 12 weeks to 24 weeks postpartum.

Three-day infant-feeding diaries [5] were collected at 6 ± 2 weeks, 12 ± 1 weeks and 24 ± 1 weeks postpartum. Participants recorded the time breastfeeding started so that CGM glucose data from CareLink (https://carelink-trials.medtronic.com; accessed 20 November 2023) could be related to timing of breastfeeding. Time of breastmilk pumping data were not collected.

As previously published, all participants were advised to reduce insulin-pump dosing 1–2 h prior to childbirth. Participants were informed that some women with type 1 diabetes report hypoglycaemia with breastfeeding and, should they experience this, that a small carbohydrate snack during breastfeeding may be consumed. Use of the SmartGuard ‘Suspend-before-low’ feature, which stops insulin delivery when approaching a low CGM glucose level, was permitted among participants randomised to sensor-augmented pump therapy; three participants were advised to use this feature but only one did so [7].

Information on carbohydrate intake and use of open-loop or closed-loop therapy during breastfeeding was obtained from CareLink. Our analysis focused on nighttime (23:00–07:00 hours) breastfeeding. In doing so, the relationship between breastfeeding and maternal CGM glucose levels was explored during a period when factors that strongly influence maternal glycaemia (i.e. meals and exercise) would be infrequent. To allow comparison with other studies [8, 9], we determined the incidence of low glucose, defined as CGM glucose <3.9 mmol/l, of any duration in the 3 h after the initiation of breastfeeding.

The University of Calgary’s (Calgary, AB, Canada) Conjoint Health Research Ethics Board (Ethics ID: REB19–1470) and the three other Canadian recruitment centres (Mount Sinai Hospital, Toronto, ON; Laval University, Quebec City, QC; and University of Manitoba, Winnipeg, MB) approved this study. All participants signed written informed consent.

### Statistical analysis

**Fig. 1:**
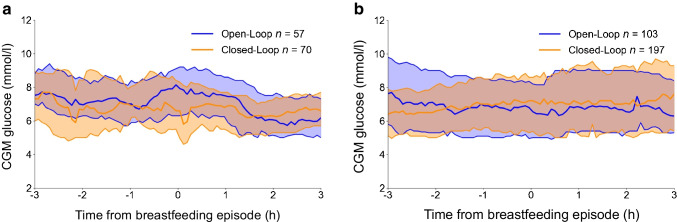
Glycaemic response to (**a**) nighttime breastfeeding (open-loop therapy, *n*=57 breastfeeding episodes; closed-loop therapy, *n*=70 breastfeeding episodes) and (**b**) daytime breastfeeding (open-loop therapy, *n*=103 breastfeeding episodes; closed-loop therapy, *n*=197 breastfeeding episodes). Data are presented as medians (central solid lines) and interquartile ranges (shaded areas); blue, open-loop therapy; orange, closed-loop therapy. In (**a**), *p*<0.01 for decreased in blood glucose within 3 h of the initiation of breastfeeding with use of closed-loop vs open-loop therapy

CGM glucose data 3 h pre- and post-breastfeeding were compared according to the insulin delivery method used at the time of the breastfeed (i.e. open-loop vs closed-loop). Generalised mixed models were adjusted for breastfeeding episodes in the same woman, repeat breastfeeding episodes, carbohydrate intake, infant age at time of feed and early pregnancy HbA_1c_. Breastfeeding episodes were considered repeated if breastfeeding occurred more than once in the 3 h after initiation of breastfeeding. We pre-specified the use of separate models for breastfeeding episodes that occurred at nighttime (23:00–07:00 hours) and daytime (07:01–22:59 hours). Data for CGM glucose 3 h pre- and post-breastfeeding are presented as medians and interquartile ranges (Fig. 1). We determined the incidence of low CGM glucose after breastfeeding and compared this between each insulin delivery method.

All statistical analyses were performed using Stata 17 (StataCorp, College Station, TX, USA). A two-sided *p* value of <0.05 was considered significant.

## Results

In total, 17 of 18 (94%) participants breastfed or pumped breastmilk for varying durations in the postpartum period [7]. Baseline characteristics are shown in Table 1. The mean age for participants was 32.0 ± 3.4 years, with a mean duration of diabetes of 21.9 ± 7.2 years.

**Table 1:** Characteristics of study participants

Characteristic	Value
Participants, *n*	18
Age at time of randomisation, years	32.0 ± 3.4
Diabetes duration, years	21.9 ± 7.2
Pre-pregnancy BMI, kg/m^2^	26.2 ± 4.1
Pre-pregnancy weight, kg	71.2 ± 12.1
HbA_1c_ in early pregnancy, mmol/mol^a^	51.0 ± 7.1
HbA_1c_ in early pregnancy, %^a^	6.91 ± 0.89
Highest level of education	
Undergraduate degree	8 (44.4)
University degree beyond undergraduate degree	6 (33.3)
Trade or technical school	4 (22.2)
Self-reported race^b^	
European/Mediterranean origin	15 (83.3)
South Asian origin	1 (5.6)
None of the options for race apply	2 (11.1)
Self-reported gender, woman	18 (100)
Gestational age at delivery	37.1 ± 1.8
Primiparous	9 (50.0)
Type of delivery	
Vaginal birth	9 (50.0)
Caesarean birth	9 (50.0)
Neonatal outcomes	
Neonatal hypoglycaemia	7 (38.9)
Neonatal intensive care unit admission	3 (16.7)

Data are mean ± SD or *n* (%), unless otherwise indicated

^a^HbA_1c_ in early pregnancy was determined between 5 weeks’ and 15 weeks’ gestation

^b^Participants were given the following options for race: Black origin (e.g. Caribbean or any of the original peoples of Africa, etc.); East Asian origin (e.g. Far East Asia, etc.); European/Mediterranean origin (e.g. Europe [including Spain] and Western Russia etc.); Hispanic origin (e.g. Central and South America, etc.); Indigenous (e.g. First Nations, Indian, Inuit or Métis people, Native Americans etc.); Jewish origin (Ashkenazi or Sephardic, etc.); Middle Eastern origin (e.g. Afghanistan, Arab, Bedouin, Iran, etc.); Pacific Island origin (e.g. Samoa, Hawaii, etc.); South Asian origin (e.g. Indian subcontinent, etc.); multi-ethnic (two or more ethnicities); unknown/not reported; or none of the above apply

Overall, infant-feeding diaries were available for 17 (94%) participants (*n*=15 at 6 weeks, *n*=16 at 12 weeks and *n*=16 at 24 weeks postpartum). Overall nighttime breastfeeding episodes while using open-loop or closed-loop therapy were accompanied by a small mean decrease in maternal glucose (1.1 - l^−1^ h^−1^; *p*=0.009) within 3 h of the initiation of breastfeeding (Fig. 1a). The decrease in glucose with nighttime breastfeeding was less with closed-loop therapy (0.4 ± 0.3 mmol/l) compared with open-loop therapy (1.2 ± 0.5 mmol/l) (mean difference: 0.8 mmol/l [95% CI 0.73, 0.88]; *p*<0.01). Daytime breastfeeding did not result in a significant mean glucose decrease within 3 h of the initiation of breastfeeding (Fig. 1b).

Of the total number of nighttime breastfeeding episodes (*N*=127), six (5%) were accompanied by carbohydrate ingestion, as reported in the pump record 30 min before or after a breastfeeding episode (*n*=3 in the open-loop therapy group and *n*=3 in the closed-loop therapy group).

The number of breastfeeding episodes is shown in Table 2, along with incidence of low glucose in the 3 h after breastfeeding initiation. Overall, glucose remained at or above 3.9 mmol/l in 93% (397) of the 427 breastfeeding episodes within the 3 h after initiation of breastfeeding. The number of nighttime breastfeeding episodes with reported low glucose was 5 of 57 (8.8%) breastfeeds in the open-loop group and 2 of 70 (2.9%) breastfeeds in the closed-loop group; this difference did not reach statistical significance. For daytime breastfeeding episodes, 9 of 103 (8.7%) and 14 of 197 (7.1%) breastfeeds were followed by low glucose with open-loop and closed-loop therapy, respectively (Table 2). Nighttime glucose levels <3.9 mmol/l occurred, on average, 5.0 h and 6.4 h after a prior insulin bolus for the open-loop and closed-loop therapy groups, respectively.

**Table 2:** Maternal hypoglycaemia metrics before and after initiation of breastfeeding by time of day and insulin delivery method

Variable	Nighttime		Daytime	
	Open-loop	Closed-loop	Open-loop	Closed-loop
Women with observed feeds, *n*	4	12	4	12
Observed feeds, *n*	57	70	103	197
Repeat breastfeeding episodes, *n*^a^	3	3	22	59
Overall CGM glucose data				
Mean glucose 1 h before breastfeeding, mmol/l	7.3 ± 0.1	7.1 ± 0.1	7.1 ± 0.1	7.1 ± 0.1
Average glucose 3 h after breastfeeding, mmol/l	6.4 ± 0.2	8.1 ± 0.2	7.1 ± 0.1	7.1 ± 0.1
Mean daily glucose on logged breastfeeding days, mmol/l	7.1 ± 2.4	7.5 ± 2.9	7.1 ± 2.4	7.5 ± 2.9
Mean daily carbohydrate intake on logged breastfeeding days, g	225.8 ± 23.9	216.4 ± 25.4	225.8 ± 23.9	216.4 ± 25.4
Glucose <3.9 mmol/l				
Women with glucose <3.9 mmol/l, *n* (%) of women	4 (100)	2 (16.7)	3 (75.0)	8 (66.7)
Incidence of glucose <3.9 mmol/l for any duration, *n* (%) of observed feeds	5 (8.8)	2 (2.9)	9 (8.7)	14 (7.1)
Incidence of glucose <3.9 mmol/l lasting ≥15 min, *n* (%) of observed feeds	4 (7.0)	2 (2.9)	7 (6.8)	10 (5.1)
Overall time with glucose <3.9 mmol/l on logged breastfeeding days, %	5.7 ± 2.9	1.8 ± 0.8	5.7 ± 2.9	1.8 ± 0.8

Data were derived from infant-feeding diaries performed from 6 ± 2 weeks, 12 ± 1 weeks and 24 ± 1 weeks postpartum

Data are presented at mean ± SD, unless otherwise indicated

^a^Defined as more than one breastfeed in the 3 h following initiation of a breastfeeding episode

Only two of the 12 women that contributed data for nighttime breastfeeding episodes in the closed-loop therapy group experienced low CGM glucose in the 3 h following a nighttime breastfeeding episode (each on one occasion). All four women that provided data for nighttime breastfeeding in the open-loop therapy group experienced low CGM glucose in the 3 h following nighttime breastfeeding initiation. There were fewer infant-feeding episodes in the open-loop therapy vs closed-loop therapy group because open-loop therapy was only used by half the participants in the first half of the study. There were only 26 nighttime bottle-feeding episodes, none of which were followed by low glucose within 3 h.

## Discussion

CGM glucose remained above 3.9 mmol/l in the 3 h after starting breastfeeding in 93% of breastfeeding episodes. Nighttime but not daytime breastfeeding episodes were accompanied by a small decrease in maternal glucose within 3 h of the initiation of breastfeeding. The glucose decrease following nighttime breastfeeding was blunted with the use of closed-loop therapy compared with open-loop therapy.

Low CGM glucose was observed in only 2.9% of nighttime breastfeeding episodes with use of closed-loop therapy and 8.8% of nighttime breastfeeding episodes with use of open-loop therapy. Reassuringly, the low incidence of nocturnal CGM glucose <3.9 mmol/l in the 3 h following initiation of breastfeeding did not appear dependent on carbohydrate intake with breastfeeding; carbohydrate ingestion within the 30 min prior to or after breastfeeding initiation was documented in only 5% of nighttime breastfeeding episodes, which is similar to another study [8].

Milk is constantly produced in the breast tissue in breastfeeding women, so it is unsurprising that breastfeeding is accompanied by only a small decrease in maternal CGM glucose. Furthermore, decreases in glucose were only observed at nighttime, when factors that contribute to maternal glycaemic excursions, such as meals and exercise, occur infrequently. Oxytocin is secreted at the time of breastfeeding and can increase glucose uptake into skeletal muscle and cardiomyocytes [10, 11] via the oxytocin receptor [11]. This may provide an explanation for the small decrease in maternal glucose that was observed with nighttime breastfeeding episodes.

The small, but significant, decrease in glucose that occurred following the initiation of nighttime breastfeeding episodes is similar to that observed in another study [9]. This decrease was reassuringly small and is unlikely to be clinically relevant for breastfeeding women unless their blood glucose levels are at 4 mmol/l or less at the initiation of breastfeeding. Low CGM glucose occurred after 7% of all breastfeeding episodes in the 3 h following the initiation of breastfeeding. This is consistent with previous findings that showed a 5–14% incidence of low CGM glucose with blinded CGM in the 3 h following breastfeeding initiation [8, 9]. It is possible that women with type 1 diabetes who stop breastfeeding in the early postpartum period do so because they experience more hypoglycaemia. Since our study and the studies of others [8, 9] did not collect infant-feeding dairies until at least 4 weeks postpartum, it is possible that the study participants were less prone to hypoglycaemia. Interestingly, participants that stopped breastfeeding in our study reported that it was unrelated to glycaemia (M. Quintanilha, Division of Human Nutrition, University of Alberta, Edmonton, AB, Canada, personal communication), consistent with some of the previous literature [9] but conflicting with others [6].

To our knowledge, this is the first study to report on the influence of closed-loop therapy on maternal glucose response to breastfeeding, and to show that the nighttime glucose decrease with breastfeeding is blunted while using closed-loop therapy. Additional strengths of this study include the detailed breastfeeding data collected from free-living women postpartum. Limitations to our study include the small sample size and that we did not collect maternal dietary records and, thus, had to rely solely on carbohydrate data entered into the insulin pumps by the insulin-pump users. Given the small sample size it is unclear if our participants are representative of the larger type 1 diabetes population and if the findings will be consistent for people of all genders.

In conclusion, we observed a small decrease in maternal glucose after nighttime breastfeeding that usually does not result in maternal hypoglycaemia and appears blunted with the use of closed-loop therapy. Larger studies in more diverse populations are required to confirm these findings.

## Data Availability

Study data are available from the corresponding author by request after ethics review and approval for the proposed study is provided and approved by the CLIMB steering committee.

## Abbreviation

CGM: Continuous glucose monitoring

## Authors’ relationships and activities

LED reports in-kind donations and reduced cost for study supplies for investigator-initiated trials from Medtronic, Dexcom, Tandem Diabetes Care and Inter-analytics. JMY reports in-kind donations and reduced cost for study supplies for investigator-initiated trials from Medtronic and Abbott. DSF reports in-kind donations for study supplies for investigator-initiated trials from Medtronic, Dexcom, Tandem Diabetes Care and Inter-analytics, is on the Advisory Board for Ypsomed and reports honoraria from Sanofi and Novo Nordisk. HRM sits on the Medtronic European Scientific Advisory Board and reports receiving speaker honoraria from Dexcom, Abbott, Medtronic and Novo Nordisk, and is a member of the Editorial Board of *Diabetologia*. RJS reports research support from Novo Nordisk. PL reports speaker fees from Dexcom. All other authors declare that there are no relationships or activities that might bias, or be perceived to bias, their work.
